# Maternal Smoking in Pregnancy and Offspring Depression: a cross cohort and negative control study

**DOI:** 10.1038/s41598-017-11836-3

**Published:** 2017-10-03

**Authors:** Amy E. Taylor, David Carslake, Christian Loret de Mola, Mina Rydell, Tom I. L. Nilsen, Johan H. Bjørngaard, Bernardo Lessa Horta, Rebecca Pearson, Dheeraj Rai, Maria Rosaria Galanti, Fernando C. Barros, Pål R. Romundstad, George Davey Smith, Marcus R. Munafò

**Affiliations:** 1 MRC Integrative Epidemiology Unit (IEU) at the University of Bristol, Bristol, United Kingdom; 2 UK Centre for Tobacco and Alcohol Studies, School of Experimental Psychology, University of Bristol, Bristol, United Kingdom; 3 Population Health Sciences, Bristol Medical School, University of Bristol, Bristol, United Kingdom; 4 Federal University of Pelotas, Pelotas, Brazil; 5 Department of Medical Epidemiology and Biostatistics, Karolinska Institutet, Stockholm, Sweden; 6 Department of Public Health and Nursing, Faculty of Medicine, Norwegian University of Science and Technology (NTNU), Trondheim, Norway; 7 Norway and Forensic Department and Research Centre Bröset, St. Olav’s University Hospital Trondheim, Trondheim, Norway; 8 Department of Public Health Sciences, Karolinska Institutet, Stockholm, Sweden; 9 Centre for Epidemiology and Community Medicine, Stockholm County Council, Stockholm, Sweden; 10 Catholic University of Pelotas, Pelotas, Brazil

## Abstract

Previous reports suggest that offspring of mothers who smoke during pregnancy have greater risk of developing depression. However, it is unclear whether this is due to intrauterine effects. Using data from the Avon Longitudinal Study of Parents and Children (ALSPAC) from the UK (N = 2,869), the Nord-Trøndelag health study (HUNT) from Norway (N = 15,493), the Pelotas 1982 Birth Cohort Study from Brazil (N = 2,626), and the Swedish Sibling Health Cohort (N = 258 sibling pairs), we compared associations of maternal smoking during pregnancy and mother’s partner’s smoking during pregnancy with offspring depression and performed a discordant sibling analysis. In meta-analysis, maternal smoking during pregnancy was associated with higher odds of offspring depression (OR 1.20, 95% CI:1.08,1.34), but mother’s partner’s smoking during pregnancy was not (OR 1.05, 95% CI:0.94,1.17). However, there was only weak statistical evidence that the odds ratios for maternal and mother’s partner’s smoking differed from each other (p = 0.08). There was no clear evidence for an association between maternal smoking during pregnancy and offspring depression in the sibling analysis. Findings do not provide strong support for a causal role of maternal smoking during pregnancy in offspring depression, rather observed associations may reflect residual confounding relating to characteristics of parents who smoke.

## Introduction

The harmful effects of maternal smoking during pregnancy on the offspring are well established, and include increased risk of pre-term birth^1^ and low birth weight^2^. However, less is known about the impact on offspring mental health outcomes. Several studies have explored internalizing and externalizing behavioural problems in childhood^3–10^. In general, there is stronger support for an association of maternal smoking during pregnancy with externalizing problems and related outcomes such as conduct disorder, than for an association with internalizing problems such as depression^3,11^. A few studies have reported associations between maternal smoking and internalizing behaviours including depression in offspring beyond childhood^12–15^, so it is possible that if there is a causal effect of prenatal tobacco exposure on depression, it does not materialise until adolescence or adulthood.

There are plausible biological mechanisms through which maternal smoking may increase risk of depression in the offspring. There is evidence from animal studies that nicotine disrupts neurodevelopment in the fetus^16^. This is likely to occur through activation of nicotine acetylcholine receptors which modulate neurotransmitter pathways^17^. *In utero* exposure to tobacco smoke is also associated with epigenetic changes, both in the placenta^18^ and in the offspring^19,20^. There is evidence that this includes epigenetic regulation of genes involved in the hypothalamic pituitary adrenocortical axis (HPA)^20^, which is involved in the body’s response to stress. Overactivity of the HPA is commonly observed in individuals with depression^21^ and has been proposed as a potential causal pathway^22^.

Isolating whether there is a causal effect of intrauterine exposure to tobacco smoking is difficult. Associations may be due to common genetic or environmental effects in mother and offspring, or to other sources of confounding such as maternal personality characteristics associated with smoking. Statistical adjustment for potential confounders is unlikely to be adequate, given that unmeasured confounders are likely to be operating, and measured confounders will be measured imprecisely^23^. For example, in the case of maternal smoking and offspring depression, it may be difficult to adequately measure parental mental health, which is likely to be associated with parental smoking^24^ and with offspring mental health^25^. This may explain why many of the associations between maternal smoking and offspring outcomes are not replicated using study designs that support stronger causal inference, such as sibling and twin studies and *in vitro* fertilization studies comparing genetically related and unrelated offspring^4,26–28^. To our knowledge, similar methodological approaches have not been used to explore the relationship between maternal smoking during pregnancy and offspring depression.

One further approach is to include a negative control exposure in the analysis, where no association or an association of much smaller magnitude would be expected^29^ for the negative control exposure if the primary exposure variable is causal. In the case of maternal smoking during pregnancy, this can be done by comparing associations of maternal and the mother’s partner’s smoking during pregnancy with offspring outcomes. Given that only mothers are connected biologically to the fetus, if there were an intrauterine effect of tobacco exposure, we would expect associations between maternal smoking and offspring outcomes to be stronger than associations between partner smoking and offspring outcomes^30^. If effects are of similar magnitude, this suggests that associations between maternal smoking during pregnancy and offspring outcomes are more likely to be due to confounding, either by shared environmental or genetic factors, which may themselves be causal factors, or due to a causal influence operating outside of pregnancy, such as parental behavioural influences in childhood and adolescence^31^. Cross-cohort comparison can also provide stronger evidence for causality; associations are more likely to be causal if they are seen across populations with different confounding structures^5^. Finally, another approach that strengthens causal inference is to compare siblings discordant for exposure to maternal smoking during pregnancy, which partially controls for genetic and environmental confounding^26,32^. It is important to triangulate results from different study designs; each of these methods has different strengths and weaknesses, so it is unlikely that any single study will provide a definitive answer. For example, discordant sibling studies may be less subject to bias from confounding, but often provide imprecise results due to low power^33^. However, if these different methods provide converging evidence, that is more compelling.

In this study we applied the analytical methods described above on data from three population based cohort studies from three different countries and a sibling study from a fourth country to assess whether maternal smoking during pregnancy is likely to be a causal risk factor for offspring depression during late adolescence and adulthood.

## Results

The numbers of individuals contributing to the analyses from each study were: 2,869 in ALSPAC, 15,493 in HUNT and 2,626 in Pelotas 1982 when we restricted analyses to individuals with complete information on confounders (Table 1). Offspring depression was measured at age 17 years (SD 0.4) in ALSPAC, 32 years (SD 8.6) in HUNT and 30 years (SD 0.1) in Pelotas 1982. Prevalence of offspring depression was between 6% and 8% in the three studies.

**Table 1:** Characteristics of study populations in negative control analysis.

	N	Mean age of offspring (SD)	Male (%)	Maternal smoking during pregnancy (%)	Partner smoking during pregnancy (%)	Depression (%)	Maternal education (>12 years) (%)	Non-manual social class (%)	Maternal age at birth of child (Mean (SD))	Maternal depression (%)
**ALSPAC**	2,869	17.8 (0.4)	44.8	14.3	29.6	6.9	50.1	66.8	29.5 (4.4)	9.6
**HUNT**	15,493	32.4 (8.6)	47.9	36.0	57.5	7.8	12.3	30.2	26.3 (5.2)	11.9
**Pelotas 1982**	2,626	30.2 (0.1)	48.4	33.2	58.6	7.7	14.6	15.6	26.5 (6.2)	N/A

In all studies, both maternal and partner smoking during pregnancy were associated with lower maternal education and social class (Supplementary material, eTables 1 and 2), apart from in HUNT where maternal smoking was not associated with social class. Within each study, patterns of associations with confounders were broadly similar for maternal and partner smoking, but did show some evidence for differences (Supplementary eFigure 5). For example, in ALSPAC, maternal smoking showed stronger associations with maternal education than partner smoking but in HUNT, partner smoking was more strongly associated with maternal education than maternal smoking. In ALSPAC, there was evidence that maternal smoking was more strongly associated with maternal depression than partner smoking. Offspring depression was associated with lower maternal education in all studies (although only weakly in ALSPAC) and with increased likelihood of maternal depression in ALSPAC and HUNT (maternal depression was not available for all individuals in Pelotas 1982), but was only associated with lower social class in Pelotas 1982.

**Figure 1:**
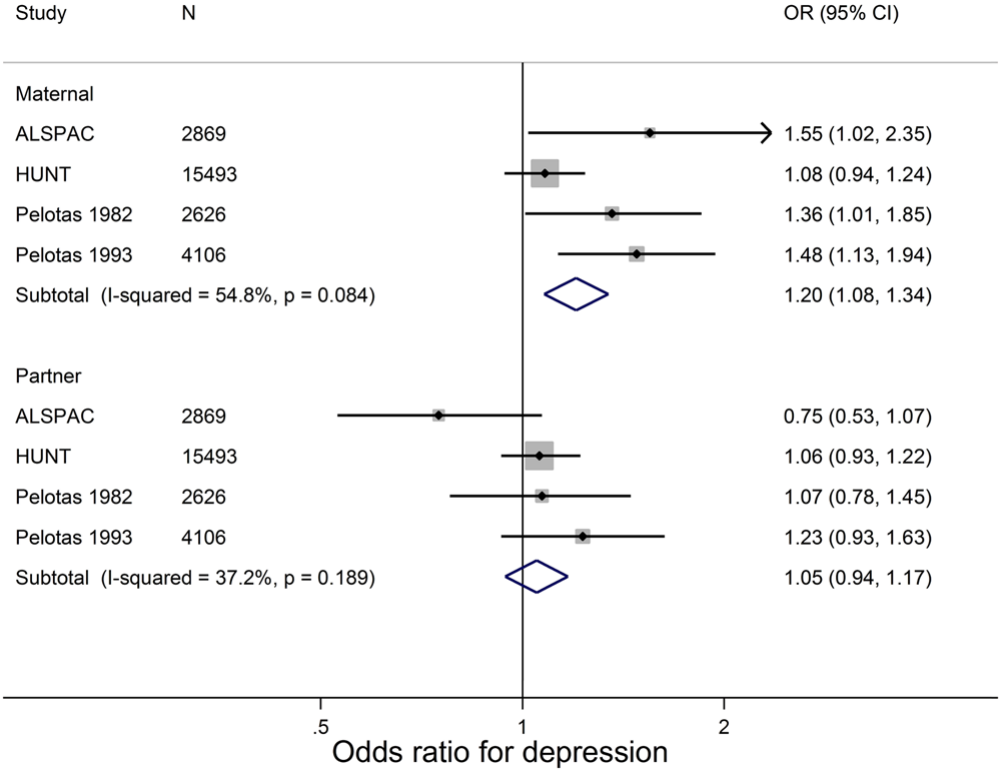
Meta-analysis of associations between parental smoking and offspring depression. Analyses adjusted for all confounders and other parent’s smoking during. Pelotas 93 results published by Menezes *et al*.^12^. In Pelotas 1993, depression was assessed at age 18. Analyses were adjusted for sex, family income at birth, planned pregnancy, partner support of pregnancy, alcohol use during pregnancy, type of delivery, partner’s smoking during pregnancy and mother’s SRQ when offspring were 11 years old.

After mutual adjustment for the other parent’s smoking (Table 2) there was evidence that maternal smoking during pregnancy was associated with higher odds of offspring depression in ALSPAC and Pelotas 1982, but no strong evidence for this association in HUNT. After adjustment, there was no clear evidence for associations between partner smoking during pregnancy and offspring depression in any of the studies. When the results of the mutually adjusted analyses were meta-analysed, together with the previously published results from the Pelotas 1993 birth cohort (Fig. 1), there was evidence that maternal smoking during pregnancy was associated with higher odds of offspring depression (OR: 1.20, 95% CI: 1.08, 1.34) but no clear evidence that partner smoking during pregnancy was associated with higher odds of offspring depression (OR: 1.05, 95% CI: 0.94, 1.17). There was only weak statistical evidence that the odds ratios for maternal and partner smoking differed from each other (P value from Cochran’s Q test = 0.08). There was evidence for moderate heterogeneity between studies (I-squared values 54% for maternal smoking, 37% for partner smoking). Multiple imputation of missing data in ALSPAC and HUNT produced similar estimates (see eTable 6).

**Table 2:** Associations between parental smoking and offspring depression.

	N	Unadjusted OR (95% CI)	P-value	Partially adjusted OR (95% CI)^1^	P-value	Fully adjusted OR (95% CI)^2^	P-value	Mutually adjusted OR (95% CI)^3^	P-value
**ALSPAC**									
Maternal smoking	2,869	1.72 (1.21, 2.46)	0.003	1.71 (1.20, 2.45)	0.001	1.39 (0.94, 2.06)	0.10	1.55 (1.02, 2.35)	0.04
Partner smoking	2,869	1.03 (0.75, 1.41)	0.87	1.01 (0.74, 1.39)	0.94	0.84 (0.60, 1.17)	0.29	0.75 (0.53, 1.07)	0.11
**HUNT**									
Maternal smoking	15,493	1.02 (0.90, 1.16)	0.71	1.23 (1.08, 1.40)	0.002	1.10 (0.96, 1.26)	0.19	1.08 (0.94, 1.24)	0.27
Paternal smoking	15,493	1.31 (1.15, 1.48)	<0.001	1.20 (1.06, 1.37)	0.005	1.08 (0.95, 1.23)	0.25	1.06 (0.93, 1.22)	0.38
**Pelotas 1982**									
Maternal smoking	2,626	1.50 (1.13, 2.02)	0.006	1.52 (1.13, 2.04)	0.005	1.35 (1.00, 1.82)	0.05	1.36 (1,01, 1.85)	0.05
Partner smoking	2,626	1.16 (0.86, 1.56)	0.32	1.24 (0.92, 1.67)	0.16	1.12 (0.83, 1.52)	0.45	1.07 (0.78, 1.45)	0.68

^1^Adjusted for offspring age and sex.

^2^Adjusted for all covariates. ALSPAC: maternal age, partner social class, maternal education, maternal and paternal antenatal depression and anxiety, parity, housing tenure, crowding. HUNT: maternal age, partner occupation, maternal education, maternal and paternal depression and anxiety at survey, parity, wave of HUNT participation, number of HUNT participations. Pelotas 1982: maternal age, social class, maternal education, household income, assets index, crowding.

^3^Adjusted for all covariates and the other parent’s smoking.

### Discordant sibling analysis

The within sibling pair analysis was conducted in 258 sibling pairs discordant for maternal smoking during pregnancy from the Swedish Sibling Health Cohort (see eTable 4 in Supplementary Material for full results). After adjustment for potential confounders, there was no clear evidence for a difference in the odds of lifetime depression between siblings exposed to maternal smoking whilst *in utero* and those not exposed (OR for depression in exposed vs unexposed: 1.03, 95% CI: 0.77, 1.36).

## Discussion

We found that maternal smoking during pregnancy but not paternal smoking during pregnancy was associated with a small increased risk of offspring depression, but that individuals whose mothers smoked during pregnancy were no more likely to have depression when compared to their siblings from another pregnancy in which the mother abstained from smoking. Taken together, these results suggest that the association between maternal smoking during pregnancy and offspring depression may be confounded by unmeasured factors. Furthermore, even if causal, the results suggest any effect is likely to be small. Disentangling causal effects from non-causal associations is notoriously difficult, particularly in the context of complex behaviours such as cigarette smoking. Here we attempted to combine multiple methods, including negative control and discordant sibling analyses, as well as a comparison across different populations and contexts.

When we meta-analysed the results of the negative control analyses, we found some evidence for association of maternal smoking during pregnancy with offspring depression after adjustments for potential confounders. These results are consistent with previous reports of associations between maternal smoking during pregnancy and offspring internalizing behaviours in adulthood and adolescence^13–15^. However, these previous studies did not use methodologies such as negative control comparisons to try to assess the impact of confounding. The lack of a clear difference between the maternal and partner estimates in the negative control analysis and the null association in the sibling analysis, which should control more comprehensively for shared familial environment, suggests that these observational associations may simply be due to confounding.

Our analyses of the confounding structures between and within our studies provide some clues as to what types of unmeasured factors might be driving the observed association between maternal smoking during pregnancy and offspring depression. Differences in the relationship between maternal and partner smoking during pregnancy and socioeconomic variables (social class and education) appeared most marked in ALSPAC, which also had the largest difference between estimates of the association of maternal and partner smoking with offspring depression. However, offspring depression was only weakly associated with social class and maternal education in ALSPAC, so it is unlikely that socioeconomic factors explain the difference between maternal and paternal estimates in this study. Furthermore, in Pelotas 1982, maternal and paternal smoking were similarly socially patterned but the point estimate for the association between maternal smoking and offspring depression was stronger than for partner smoking. In contrast, maternal smoking was much more strongly associated with maternal depression than partner smoking was in ALSPAC, suggesting that maternal specific factors such as maternal mental health could be driving this association. This might be a consequence of the fact that the likely perceived impact of smoking on the fetus is different for mothers and fathers, i.e., mothers are smoking within a context of greater perceived potential risk to the fetus than fathers smoking. Differential associations between smoking and depression are especially relevant because it has been shown previously within ALSPAC that maternal antenatal depression is associated with offspring depression, but there is no such evidence for an association with paternal antenatal depression^34^. This is thought to be because there are maternal specific causal pathways such as *in utero* effects of antenatal depression on fetal programming. Thus the maternal smoking associations may just reflect the impact of maternal antenatal depression. It is unlikely that we fully accounted for parental mental health in these analyses. In ALSPAC and HUNT, we only adjusted for parental depression and anxiety at a single time point and we were unable to adjust for these in the main analysis for Pelotas 1982. Maternal depression was self-reported by a subsample of mothers when the offspring were 18 years old in Pelotas 1982; adjustment for this did make estimates for maternal and paternal smoking very similar, but small numbers meant that both estimates were imprecise (see Supplementary eTable 7).

There are a number of potential limitations that should be considered when interpreting these results. First, where partner or paternal smoking is used as a negative control the mother may still be exposed via environmental tobacco smoke (i.e., “passive” smoking). However, we have shown that levels of exposure, as assessed by cotinine (the primary metabolite of nicotine) are low in non-smoking pregnant women where the partner smokes compared to active smoking^35^. This suggests that using mother’s partner’s smoking as a negative control for investigating intrauterine effects is valid in terms of biological effects. Second, the results may be affected by loss to follow up; in all studies the analysis sample is substantially smaller than the initial number recruited. This could lead to biased estimates if inclusion in the analysis sample is related to offspring depression and to smoking of one parent during pregnancy more than the other. Patterns of missing data in all three studies suggest that missingness is related to parental smoking patterns (see Supplementary eTable 5). In HUNT and ALSPAC, but not in Pelotas 1982, offspring with missing exposure and covariate data had higher levels of depression. However, imputation of missing data in ALSPAC and HUNT did not substantially alter results. Third, there is likely to be error in the measurement of parental smoking during pregnancy and offspring depression, which are all based on self-report. It is known that smoking during pregnancy is likely to be underreported, particularly by mothers^36^. In both ALSPAC and Pelotas mothers were asked about regular or daily smoking during pregnancy, so it is possible that occasional smokers would have been classified as non-smokers. In HUNT, both parents’ smoking during pregnancy was inferred from dates of starting and quitting so a certain degree of exposure misclassification may have occurred. Conversely, if there is stigma surrounding smoking during pregnancy, it could increase accuracy to not have a direct question about smoking during pregnancy. In Pelotas 1982, data on paternal smoking was only collected in the fathers when the offspring were age 4 years. Whilst it is unlikely that many fathers had taken up smoking in this period, it is possible that some had given up smoking and so were wrongly misclassified as non-smokers. It is likely that misclassification of maternal smoking will also have affected the results of the sibling analysis, as a proportion of these pairs are probably actually concordant for maternal smoking, but in one pregnancy smoking has been misclassified^37^. Such misclassification could potentially bias results to a large degree and in either direction^38^. Fourthly, it should be noted that the sibling analysis was only powered to detect an odds ratio of around 1.3, so we cannot completely rule out the possibility of a modest effect from this analysis. Finally, offspring depression was measured at different ages, using different scales/questions in each of the studies; whilst all measures are validated for measurement of mental health, we cannot be certain that each study is capturing the same depression phenotype. Depression is extremely heterogeneous, likely to consist of a number of subtypes, which may explain difficulties in identifying causal factors^39^. Diagnosis by a doctor may underestimate depression if individuals do not seek treatment. Similarly, using current rather than lifetime depression (which was not available in ALSPAC, Pelotas or HUNT) could also underestimate depression. This misclassification could lead to underestimation of associations between maternal smoking and offspring depression.

Although we observed some evidence for association between maternal smoking and offspring depression, the pattern of results from the negative control analysis and the sibling analysis suggest that this association is more likely to be explained by confounding by maternal specific factors than a causal effect of intrauterine exposure to tobacco smoke. Further, given the modest magnitude of the association, it is unlikely that, even if there is a true causal effect, maternal smoking during pregnancy is an important cause of offspring depression. Given the known harms of smoking during pregnancy to the developing fetus, these findings do not change health messages about the harms of smoking during pregnancy However, they add to existing knowledge about the aetiology of depression and highlight the need to use multiple analytical methods and study designs to improve causal inference when considering *in utero* exposures and disease in later life.

## Methods

### Study Information

For the analysis comparing the associations of maternal and paternal smoking with offspring depression, we used data from three studies: the Avon Longitudinal Study of Parents and Children (ALSPAC), a birth cohort of individuals from Avon in the UK (born 1992–1993)^40,41^, the Nord-Trøndelag health study (HUNT), a major population-based health study (of individuals 20 years and older) conducted in the Nord-Trøndelag county in central Norway^42^, and the Pelotas 1982 Birth Cohort^43,44^, a longitudinal birth cohort from Pelotas, a Southern Brazilian city. For the sibling analyses, we used data from the Swedish Sibling Health Cohort, a nation-wide population-based cohort study. Full details of all of these study populations are provided in supplementary material along with flowcharts of the samples contributing to these analyses (eFigures 1–4).

### Smoking during pregnancy

Information on maternal smoking during pregnancy and mother’s partner smoking during pregnancy were obtained from self-report (or report by the mother of their partner smoking) in questionnaires administered during pregnancy or just after birth of the offspring (ALSPAC, Pelotas 1982) or at the time of the cohort survey (HUNT). In Pelotas 1982, partner smoking was only assessed when the offspring were aged 4 years, so these data were used as a proxy for partner smoking during pregnancy. In HUNT, maternal and partner smoking during pregnancy were inferred from dates of smoking initiation, smoking cessation and offspring birth. In the Swedish Sibling Health Cohort, maternal smoking during pregnancy was retrieved through the Swedish Medical Birth Register, which contains information about pregnancies and deliveries for more than 98% of all births in Sweden since 1973^45^. Full details of the smoking data are provided in supplementary material.

### Depression

Depression was coded as a binary variable in all studies. In ALSPAC, current depression at age 17 was assessed by a fully structured validated instrument, the Clinical Interview Schedule – Revised (CIS-R)^46^. In HUNT, current depression was classified as scoring ≥8 on the Hospital Anxiety and Depression Scale (HADS) on at least one of up to two participation occasions^47^. In Pelotas 1982, current depression at age 30 was assessed via a diagnostic interview for major depression using the Mini-International Psychiatric Interview (MINI) version 5.0 validated for Brazil^48^. In the Swedish sibling Health Cohort, participants self-reported a lifetime history of clinical diagnosis of depression by answering the questionnaire item: “Has a physician at any time in your life told you that you had depression?”.

### Covariates

Analyses were adjusted for a number of covariates, depending on their availability in the individual studies. These included offspring age and sex, maternal age at offspring birth, social class, maternal education, maternal and partner depression and anxiety, parity, housing tenure, crowding, household income and household assets. In the discordant sibling analyses, covariates were omitted if they were invariable or almost invariable within families. A full description of the covariates is provided in Supplementary Material.

### Statistical analysis

A summary of the measures available in each study is shown in Table 3. All associations between parental smoking and offspring depression were performed within participating studies using logistic regression. Analyses were run unadjusted, adjusted for offspring age and sex, and then adjusted for all covariates. In the HUNT study, robust standard errors clustered by the identity of the parent in question (or of the mother, for the combined analyses) were used to account for the non-independence of siblings. In the Swedish Sibling Health Cohort within-family associations between maternal smoking behaviour and offspring depression were assessed using conditional logistic regression among siblings discordant for exposure and outcome. Each exposed sibling was matched to its own unexposed sibling, thereby controlling for family level covariates. These analyses were adjusted for maternal age, calendar period at birth, sibling order and parity.

**Table 3:** Description of variables used in each study.

	Smoking in pregnancy		Offspring depression	Covariates
	Mother	Partner		
**ALSPAC**	Assessed by questionnaires administered during and post pregnancy	Assessed from mother and partner questionnaires administered during and post pregnancy	Self- completed Clinical Interview Schedule-Revised (CIS-R) at 17 years	Age, sex, maternal age, partner social class, maternal education, maternal antenatal depression and anxiety, paternal depression and anxiety during pregnancy, parity, housing tenure, crowding
**HUNT**	Pregnancy smoking inferred from dates of initiation and cessation and offspring birth date	Pregnancy smoking inferred from dates of initiation and cessation and offspring birth date	Hospital Anxiety and Depression Scale (HADS)	Age, sex, maternal age, partner occupation, maternal education, maternal and paternal depression and anxiety at survey, parity, wave of HUNT participation
**Pelotas 1982**	Assessed by questionnaire at birth	Self-report at 4 years (not available during pregnancy)	Diagnostic interview for major depression (MINI) at 30 years﻿	Age, sex, maternal age, social class, maternal education, household income, assets index, crowding
**Swedish Sibling Health Cohort**	From Medical Birth Register and self-reported at first ante-natal care visit	Not available	Self-report of lifetime history of clinical diagnosis of depression	Maternal age, calendar period at birth, sibling order, parity

In studies with information available on both maternal and partner smoking during pregnancy (ALSPAC, HUNT) (or 4 years post pregnancy in the case of partner smoking in Pelotas 1982), we compared associations of maternal smoking during pregnancy (any vs none) with offspring depression to those of partner smoking during pregnancy (any vs none) with offspring depression. In addition, further analyses were run mutually adjusting for the other partner’s smoking status. We combined mutually adjusted odds ratios for associations of parental smoking during pregnancy and offspring depression from ALSPAC, HUNT and Pelotas 1982 and published data from the Pelotas 1993 birth cohort^12^ in a fixed effects meta-analysis using the metan command in Stata. Differences between combined estimates for maternal and partner smoking were assessed using Cochran’s Q statistic. Analyses were conducted in Stata (StataCorp LP, College Station TX USA) and SAS (SAS Institute Inc., Cary NC USA).

To investigate the potential impact of missing data due to loss to follow up, we performed multiple imputation in ALSPAC and HUNT using the mi impute command in Stata. Further details of the imputation methods are provided in Supplementary Material.

### Data availability

The data that support the findings of this study are available from ALSPAC, Pelotas and HUNT but restrictions apply to the availability of these data, which were used under license for the current study, and so are not publicly available. Data can be obtained in the following ways. Data used for this submission will be made available on request to the ALSPAC executive committee (alspac-exec@bristol.ac.uk). The ALSPAC data management plan (available here: http://www.bristol.ac.uk/alspac/researchers/data-access/) describes in detail the policy regarding data sharing, which is through a system of managed open access. Data were provided by the HUNT Research Centre (hunt@medisin.ntnu.no) in accordance with their regulations. Data requests can be made through an application process on the HUNT Biobank (details at https://www.ntnu.edu/hunt/data). Data used from the Pelotas Study for this submission will be made available on request to the Pelotas Data Access Committee (cpublicacoes.coortespelotas@gmail.com). The Pelotas data access information (http://www.epidemio-ufpel.org.br/site/content/studies/formularios.php) describes data availability. Permission for the sharing of individual data from the SSHC was not obtained according to the ethical rules at the time of the study.

## Electronic supplementary material

Supplementary material
