# Supplementary material for: Maternal Smoking in Pregnancy and Offspring Depression: a cross cohort and negative control study
Source: Sci Rep. 2017 Oct 3;7:12579. doi: 10.1038/s41598-017-11836-3 (PMC5626710; doi:10.1038/s41598-017-11836-3)
Supplement: Supplementary file 1 — Supplementary material [file 41598_2017_11836_MOESM1_ESM.doc]

**Maternal Smoking in Pregnancy and Offspring Depression: a cross cohort and negative control study**

Amy E. Taylor PhD* ^1,2^, David Carslake PhD ^1,3^, Christian Loret de Mola PhD ^4^, Mina Rydell PhD ^5^, Tom I.L. Nilsen PhD ^6^, Johan H. Bjørngaard PhD ^6,7^, Bernardo Lessa Horta PhD ^4^, Rebecca Pearson PhD ^3^, Dheeraj Rai PhD ^3^, Maria Rosaria Galanti PhD ^8,9^, Fernando C. Barros PhD ^10^, Pål R. Romundstad PhD ^6^, George Davey Smith MD ^1,3^,

Marcus R. Munafò PhD ^1,2^

1. MRC Integrative Epidemiology Unit (IEU) at the University of Bristol, Bristol, United Kingdom.

2. UK Centre for Tobacco and Alcohol Studies, School of Experimental Psychology, University of Bristol, Bristol, United Kingdom.

3. Population Health Sciences, Bristol Medical School, University of Bristol, Bristol, United Kingdom.

4. Federal University of Pelotas, Pelotas, Brazil.

5. Department of Medical Epidemiology and Biostatistics, Karolinska Institutet, Stockholm, Sweden

6. Department of Public Health and Nursing, Faculty of Medicine, Norwegian University of Science and Technology (NTNU), Trondheim, Norway.

7. Norway and Forensic Department and Research Centre Bröset, St. Olav’s University Hospital Trondheim, Trondheim, Norway.

8. Department of Public Health Sciences, Karolinska Institutet, Stockholm, Sweden.

9. Centre for Epidemiology and Community Medicine, Stockholm County Council, Stockholm, Sweden

10. Catholic University of Pelotas, Pelotas, Brazil.

**ALSPAC**

**Study population.** The Avon Longitudinal Study of Parents and Children (ALSPAC) is a UK longitudinal birth cohort, which recruited 14,541 pregnant women resident in Avon, UK with expected dates of delivery between 1st April 1991 and 31st December 1992. Of the 14,062 live births in the study, 13,988 children were alive at 1 year of age. Detailed information on these children, their mothers and partners have been collected since birth through questionnaires and at clinic visits. Further information on the study recruitment and data collected has been published previously ^1,2^. Please note that the study website contains details of all the data that is available through a fully searchable data dictionary: http://www.bris.ac.uk/alspac/researchers/data-access/data-dictionary/. Only one member of each twin pair or sibling (where a mother had more than one pregnancy during the study period) was included in the analysis. See Figure S1 for a flowchart of the study population contributing to these analyses.

**Mother and partner smoking during pregnancy.** Maternal smoking during pregnancy was assessed via self-report in questionnaires administered at 18 weeks gestation, 32 weeks gestation and 8 weeks post-delivery (which asked about smoking during the last 2 months of pregnancy). In addition, we used data on smoking status from a questionnaire administered between 6 and 40 weeks gestation. A full list of the smoking questions in each questionnaire is provided in the online supplementary material. Mothers who reported smoking cigarettes, cigars, pipes or other types of tobacco at any stage of pregnancy were defined as pregnancy smokers. Mothers reporting not smoking in all these questionnaires were defined as non-smokers. If mothers had at least one report of not smoking but had missing data for the rest of the questionnaires, they were defined as non-smokers only if they reported not being a regular smoker just prior to pregnancy (at the questionnaire administered at 18 weeks). Partner smoking during pregnancy was assessed by self-report in questionnaires administered at 18 weeks gestation and 8 weeks post-delivery and by mother’s report of partner smoking in questionnaires administered at 18 weeks gestation and 8 weeks post-delivery. Partners were defined as smokers during pregnancy if they or the ALSPAC mother reported them to be a smoker (of cigarettes, cigars, pipes or other) in any of the questionnaires. Partners were classified as non-smokers if they did not report being a smoker at any of the time points. If partners were missing data for some of the time points, they were classified as a non-smoker if all of their reports indicated that they were non-smokers.

**Offspring depression.** Depression was assessed by a fully structured validated instrument the Clinical Interview Schedule – Revised (CIS-R) ^3^, that was self-completed when the offspring were age 18 years. Depression was defined as a binary variable (yes/no), according to ICD-10 criteria for diagnosing depression.

**Covariates.** Analyses were adjusted for a number of covariates. Information on partner social class (1, 2, 3 non manual, 3 manual, 4, 5), educational attainment of the mother (CSE, Vocational, O level, A level, Degree), housing tenure (Mortgaged/Owned, Council, Private Rent, Other), crowding, parity (0,1,2,3,4+) and maternal age at gestation were collected in questionnaires administered during pregnancy. Social class was defined according to mother’s report of their partner’s occupation. Household crowding was calculated as the number of individuals living in the household divided by the number of rooms and categorised into four groups: 0-0.5, >0.5-0.75, >0.75-1.0, >1.0. Social class, educational attainment, housing tenure and crowding and parity were all treated as categorical in the analysis. Maternal age at offspring birth and offspring age were treated as continuous variables. Depression and anxiety in the mothers and their partners were assessed by the Edinburgh Postnatal Depression Scale (EPDS) ^4^ and the anxiety scale of the Crisp Crown Experiential Index ^5^ respectively, in questionnaires administered at 18 weeks gestation. Both of these variables were treated as continuous symptom scales in the analysis.

**HUNT**

**Study population.** The HUNT Study is a major population-based health study conducted in Nord-Trøndelag, a rural county in central Norway with a population of about 130,000. At each of three waves (HUNT1, 1984-1986; HUNT2, 1995-1997 and HUNT3, 2006-2008), every resident of at least 20 years old was invited to participate. Detailed health-related information on each participant was obtained through questionnaires and a clinic visit, and family relationships among participants were obtained by linkage to national birth data. Full details of the study are available on the HUNT website (http://www.ntnu.no/hunt). We initially extracted all 45,988 participants (hereafter, offspring) in HUNT2 or HUNT3 with at least one identified parent who participated in at least one HUNT wave. See eFigure 2 for a flowchart of the study population contributing to these analyses.

**Mother and partner smoking during pregnancy.** The questions asked at each HUNT wave included sufficient information to infer the dates of smoking initiation and/or cessation to the nearest year. This information, together with the parent's smoking status at survey and the offspring's date of birth, was used to infer whether or not each parent was a current smoker at any time during the pregnancy. A full list of the smoking questions in each questionnaire is provided in the online supplementary material.

**Offspring depression.** The questionnaires completed by participants in HUNT2 and HUNT3 included the Hospital Anxiety and Depression Scale (HADS) ^6^. This includes seven depression-related questions, each answered on a scale of 0 to 3, giving a total score of 0-21. Cases of depression were defined using a threshold of ≥8 ^6^. Participants were excluded from analysis if the answers to four or more of the seven questions were missing. If 1-3 answers were missing, cases were defined from the non-missing data, which were weighted according to each question's mean contribution to the total score. In 6,235 of the 15,493 offspring analysed, depression scores were available from both HUNT2 and HUNT3 and they were defined as cases if they were depressed on either occasion. Offspring were 32.4 (SD 8.6) years old on average at participation.

**Covariates.** Analyses were adjusted to two levels. Partial adjustment comprised the offspring's age (cubic spline with five knots, placed at the percentiles recommended by Harrell ^7^), their sex, the number of depression scores available and the HUNT wave in which their depression score was measured. Full adjustment additionally included the mother's age at the offspring's birth (linear), the partner's employment category (never/unskilled, skilled/driver, clerical worker, professional, management, farmer/fisher, self-employed businessperson), the mother's educational level (<10 years, 10-12 years, >12 years), the mother's and father's depression scores and anxiety scores (binary), the offspring's smoking status at age 17 (binary; inferred in the same way as parental status during pregnancy) and the offspring's birth order among the mother's participating offspring (1^st^, 2^nd^, 3^rd^ or at least 4^th^). Offspring covariates were taken from the same HUNT wave as offspring depression, or from HUNT2 when two measurements of depression were available. Parental covariates were taken from the same HUNT wave as parental smoking, except that parental depression and anxiety scores were taken from the earliest available HUNT wave (since depression and anxiety were not recorded in HUNT1, from which most parental exposure data were taken). Robust standard errors clustered by the identity of the parent in question (or of the mother, for the combined analyses) were used to account for the non-independence of siblings.

**Pelotas 1982**

**Study population.** The 1982 Pelotas Birth Cohort Study is a Brazilian longitudinal birth cohort. Between 1st January and 31st December 1982, all maternity hospitals located in Pelotas, a southern Brazilian city, were visited daily and all newborns identified, representing 99.2% of the total births in the city. The 5,914 liveborns whose families lived in the urban area of the city were examined and their mothers interviewed. These individuals have been followed in several occasions, information on the study recruitment and data collected has been published previously^8-10^. See Figure S3 for a flowchart of the study population contributing to these analyses.

**Mother and partner smoking during pregnancy.** Maternal smoking during pregnancy was evaluated at in a questionnaire administered at birth. Mothers were asked if they had smoked during pregnancy and the number of cigarettes consumed per day. A full list of the smoking questions in each questionnaire is provided in the online supplementary material. Mothers who reported smoking at least one cigarette per day, at any stage of pregnancy, were defined as pregnancy smokers. No data was collected on partner smoking at birth, so partner smoking when the offspring were 4 years old was used in the analysis. A full list of the smoking questions in each questionnaire is provided in the online supplementary material.

**Offspring depression.** From June 2012 to February 2013, at a mean age of 30.2 years, 3,701 of the cohort were interviewed again. In this follow-up, a psychological interview was carried out, including a diagnostic interview for major depression using the Mini-International Psychiatric Interview (MINI) version 5.0 validated for Brazil ^11^. We considered a person as having major depression if he or she presented an episode of major depression during the last two weeks and did not report a manic/hypomanic episode during life, according to the MINI.

**Covariates.** Analyses were adjusted for a number of covariates. Information on household social class (1, 2, 3 non manual, 3 manual, 4, 5), maternal years of schooling, household income at birth, assets index, crowding, parity and maternal age at birth were collected in questionnaires administered at baseline and childhood. Social class was defined according to mother’s report of their occupation and their partner’s occupation. Household social class was the highest category of mother’s own and partner’s social class. Household crowding was calculated as the number of individuals living in the household divided by the number of rooms.

**Swedish Sibling Health Cohort (Syskonhälsa).**

**Study population.** The Swedish Sibling Health Cohort is a population-based cohort study conducted in Sweden with the primary scope to analyse exposure to maternal smoking during pregnancy in relation to offspring’s later smoking behaviour and tobacco dependence. In this study a nation-wide sample of same-sex full siblings born 1983-1991, discordant for maternal smoking during pregnancy was identified through computerized population and health-care registers, and constituted the study population. Of the random sample of 5,000 eligible sibling pairs, 1,538 complete pairs participated in the study. Details about the recruitment and the data collection have been published previously ^12^. Between October 2010 and March 2011 the consenting individuals were reached for a questionnaire-based survey, aimed to assess health status, perceived health, family characteristics during childhood and adolescence, own use of tobacco, alcohol and drugs**.** This analysis included only sibling pairs who were discordant for both maternal smoking and offspring depression. See Figure S4 for a flowchart of the study population contributing to these analyses.

**Mother smoking during pregnancy.** Maternal smoking during pregnancy was retrieved through the Medical Birth Register, which contains information about pregnancies and deliveries for more than 98% of all births in Sweden since 1973 ^13^. The information on smoking stored in the register was self-reported by expecting mothers to midwives at the first ante-natal care visit (usually between the 8th and 12th gestational week), registered in the midwife record and subsequently computerized. This information was available from 1983, therefore for all birth cohorts represented in this study, categorised as: no daily smoking; smoking 1–9 cigarettes per day; or smoking ≥10 cigarettes per day. No prospectively collected information was available about mother’s partner smoking habits at the time of the index pregnancy. A full list of the smoking questions in each questionnaire is provided in the online supplementary material.

**Offspring depression.** Participants self-reported a lifetime history of clinical diagnosis of depression by answering the questionnaire item: “Has a physician any time in your life told you that you had depression?”.

**Covariates.** Since the siblings were matched for sex and for biologic parents, they were inherently matched also for most of the covariates linked to family circumstances. A number of covariates differing between matched siblings were used to adjust for potential confounding in the statistical analysis, such as calendar period at birth, sibling order; maternal age and parity at the index birth, assessed through the Medical Birth Register.

**Questions on smoking during pregnancy**

**ALSPAC**

**Maternal smoking (questionnaires answered by mothers)**

**Questionnaire A (administered between 8 and 40 weeks gestation)**

*How many cigarettes a day do you smoke at the moment?*

*Answer given as continuous number*

**Questionnaire B (administered at 18 weeks gestation)**

*Have you ever been a smoker?*

*Yes*

*No*

*Did you smoke regularly before pregnancy (in the last 9 months)?*

*No*

*Yes, cigarettes*

*Yes, cigars*

*Yes, pipe*

*Yes, other (please describe)*

*Did you smoke regularly in the first 3 months of pregnancy?*

*No*

*Yes, cigarettes*

*Yes, cigars*

*Yes, pipe*

*Yes, other (please describe)*

*Did you smoke regularly in the last 2 weeks?*

*No*

*Yes, cigarettes*

*Yes, cigars*

*Yes, pipe*

*Yes, other (please describe)*

*How many times per day did you smoke at each of these times? (0, 1-4, 5-9, 10-14, 15-19, 20-24, 25-29, 30+)*

**Questionnaire C (administered at 32 weeks gestation)**

*How many cigarettes per day are you yourself smoking at the moment?*

*Answer given as continuous number*

**Questionnaire E (administered at 8 weeks post birth)**

*Did you smoke cigarettes regularly in the last 2 months of pregnancy?*

*Yes*

*No*

*Did you smoke cigars regularly in the last 2 months of pregnancy?*

*Yes*

*No*

*Did you smoke a pipe regularly in the last 2 months of pregnancy?*

*Yes*

*No*

*Did you smoke other tobacco products regularly in the last 2 months of pregnancy?*

*Yes*

*No*

*How many cigarettes (pipes or cigars) per day did you smoke in the last 2 months of pregnancy? (0, 1-4, 5-9, 10-14, 15-19, 20-24, 25-29, 30+)*

**Partner smoking during pregnancy (questionnaires answered by mothers)**

**Questionnaire B (administered at 18 weeks gestation)**

*Does your partner smoke?*

*No*

*Yes, cigarettes*

*Yes, cigars*

*Yes, pipe*

*Yes, other (please describe)*

*Don't have a partner*

*About how many times per day does your partner smoke at the moment?* *(0, 1-4, 5-9, 10-14, 15-19, 20-24, 25-29, 30+)*

**Questionnaire E (administered at 8 weeks post birth)**

*How many cigarettes (pipes or cigars) per day did your partner smoke in the last 2 months of pregnancy?*

*(0, 1-4, 5-9, 10-14, 15-19, 20-24, 25-29, 30+)*

**Partner smoking during pregnancy (questionnaires answered by partners)**

**Questionnaire PB (administered at 18 weeks gestation)**

*Have you ever been a smoker?*

*Yes*

*No*

*How many times per day did you smoke at the start of your partner’s pregnancy? (0, 1-4, 5-9, 10-14, 15-19, 20-24, 25-29, 30+)*

*How many times per day did you smoke in the last 2 weeks? (0, 1-4, 5-9, 10-14, 15-19, 20-24, 25-29, 30+)*

**Questionnaire PC (administered at 8 weeks post birth)**

*Did you smoke cigarettes regularly in the last 2 months of your partner’s pregnancy?*

*Yes*

*No*

*Did you smoke cigars regularly in the last 2 months of your partner’s pregnancy?*

*Yes*

*No*

*Did you smoke a pipe regularly in the last 2 months of your partner’s pregnancy?*

*Yes*

*No*

*Did you smoke other tobacco products regularly in the last 2 months of your partner’s pregnancy?*

*Yes*

*No*

*How many cigarettes (pipes or cigars) per day did you smoke in the last 2 months of your partner’s pregnancy? (0, 1-4, 5-9, 10-14, 15-19, 20-24, 25-29, 30+)*

**HUNT (translated from the original Norwegian)**

**Smoking in mothers, fathers and offspring - questionnaires completed at HUNT participation**

**HUNT1**

*Do you currently smoke daily?*

*If yes, do you smoke cigarettes daily?*

*If yes, do you smoke a pipe daily?*

*If yes, do you smoke cigars (or cheroots/cigarillos) daily?*

*If you do not currently smoke cigarettes daily, have you ever smoked cigarettes daily?*

*If yes, how long ago did you stop smoking cigarettes daily?*

*If you currently smoke cigarettes daily or have previously done so:*

*How many cigarettes do you smoke or did you smoke a day? (give the number per day, including hand rolled cigarettes)*

*How old were you when you started smoking daily?*

*How many years in total have you smoked daily?*

**HUNT2**

*Do you smoke?*

*Yes, cigarettes daily*

*Yes, cigar/cigarillos daily*

*Yes, pipe daily*

*Never smoked daily*

*If you currently or previously smoked daily:*

*How many cigarettes do you or did you usually smoke daily?*

*How old were you when you started smoking?*

*If you ever smoked previously:*

*How long has it been since you stopped? (Number of years)*

**HUNT3**

*Do you smoke?*

*No, I have never smoked*

*No, I have quit smoking*

*Yes, cigarettes occasionally (parties/vacation, not daily)*

*Yes, cigarettes daily*

*Yes, cigars/cigarillos/pipe daily*

*If you currently or previously smoked daily:*

*How many cigarettes do/did you usually smoke daily?*

*How old were you when you started smoking daily?*

*If you previously smoked daily, how old were you when you quit smoking?*

*If you now or earlier smoked occasionally:*

*How many cigarettes do/did you usually smoke in a month?*

*How old were you when you started smoking occasionally?*

*If you previously smoked occasionally, how old were you when you quit?*

**Pelotas 82**

**Maternal smoking (questionnaires answered by mothers)**

**Questionnaire administered at birth**

*How many cigarettes per day did you smoke during pregnancy and did you smoke during all pregnancy?*

1. *No smoking*
2. *1-14 cigarettes all pregnancy*
3. *1-14 cigarettes partially*
4. *15 or more cigarettes all pregnancy*
5. *15 or more cigarettes partially*

**Swedish Sibling Health Study**

The information stored in the midwife record and subsequently in the Birth Register has the following format:

Currently smoking cigarette per day:

0 (no daily smoking)

1-9

10 or more

Since the information is not collected by questionnaire, but as part of an anamnestic interview the exact wording of the question posed by the midwife is unknown and may vary.

**Multiple imputation**

To investigate the potential impact of missing data due to loss to follow up, we performed multiple imputation in ALSPAC and HUNT using the mi impute command in Stata. In ALSPAC, the outcome and covariates were imputed in the sample with complete exposure data (using depression data collected throughout childhood) and the exposure and covariates imputed in the sample with complete outcome data. In HUNT, the outcome, exposure and covariates were imputed up to the full sample of offspring with at least one parent who participated in at least one HUNT wave. For each analysis, 100 imputed datasets were created. Full details of the variables used in the imputation are available in supplementary material.

**eFigure 1. Flowchart of ALSPAC study population**

14,062 live births in the study

9,193 with data on maternal and partner smoking during pregnancy

3,905 with depression data at age 18 years

2,869 with data on full set of confounders

**eFigure 2. Flowchart of HUNT study population**

36,249 participants in HUNT2 and 33,538 in HUNT3 (45,988 individuals; "offspring") with at least one parent who participated in at least one HUNT wave

23,301 offspring in HUNT2 and 22,871 in HUNT3 with data on maternal and paternal smoking during pregnancy

23,045 offspring in HUNT2 and 17,689 in HUNT3 with offspring depression data

12,005 offspring in HUNT2 and 9,723 in HUNT3 (15,493 individuals from 9,232 mothers and 9,225 fathers) with data on full set of confounders

**eFigure 3. Flowchart of Pelotas 1982 study population**

5,914 offspring (excluding multiple births and siblings)

4,105 with data on maternal and partner smoking during pregnancy

3,576 with depression data

2,626 with data on full set of confounders

**eFigure 4. Flowchart of Swedish Sibling Health Cohort study population**

4,368 individuals participated in the study

1,538 complete sibling pairs discordant for maternal smoking during pregnancy

1,475 sibling pairs with depression data

258 sibling pairs discordant with regard to depression

226 sibling pairs with data on full set of confounders

**eFigure 5. Associations between parental smoking and covariates in ALSPAC, HUNT and Pelotas 1982**

B

A

C

D

eFigure 5A-C show odds ratios and 95% CIs for associations between parental smoking and covariates. eFigure 5D shows mean maternal age and 95% CIs. A. Partner’s occupation is non-manual compared to manual. B. Maternal education >12 years (in ALSPAC, this is the proportion of mothers with A-levels or higher). C. In ALSPAC, mothers categorised as depressed on the Edinburgh Post-natal Depression Scale, measuredat 18 weeks gestation. In HUNT, mothers categorised as depressed at survey using the Hospital Anxiety and Depression Scale.

**eTable 1. Covariates stratified by maternal smoking during pregnancy in complete case analysis**

|  | **ALSPAC** | | | **HUNT** | | | **Pelotas 1982** | | |
| --- | --- | --- | --- | --- | --- | --- | --- | --- | --- |
|  | **Yes** | **No** | **P-value^1^** | **Yes** | **No** | **P-value^1^** | **Yes** | **No** | **P-value^1^** |
| N (%) | 411 (14.3) | 2,458 (85.7) |  | 5,577 (36.0) | 9,916 (64.0) |  | 873 (33.2) | 1753 (66.8) |  |
| Offspring age (years) | 17.8 (4.0) | 17.8 (4.0) | 0.13 | 30.3 (7.4) | 33.6 (9.0) | <0.001 | 30.2 (0.3) | 30.2 (0.3) | 0.02 |
| % male | 43.3 | 45.0 | 0.52 | 47.2 | 48.3 | 0.20 | 48.3 | 48.4 | 0.96 |
| Maternal age at birth of offspring | 27.6 (4.7) | 29.8 (4.2) | <0.001 | 25.4 (4.7) | 26.8 (5.3) | <0.001 | 25.2 (5.9) | 27.1 (6.3) | <0.001 |
| Parity | 1.7 (0.9) | 1.7 (0.8) | 0.73 | 1.7 (0.9) | 1.8 (1.0) | <0.001 | 2.6 (1.9) | 2.6 (2.0) | 0.92 |
| Social class  (% non-manual) | 49.2 | 69.7 | <0.001 | 29.7 | 30.5 | 0.41 | 13.4 | 16.7 | 0.03 |
| Maternal education  (% >12 years education)^2^ | 29.2 | 53.6 | <0.001 | 9.1 | 14.1 | <0.001 | 8.9 | 17.5 | <0.001 |
| Crowding^3^ | 5.4 | 1.7 | <0.001 | N/A | N/A | N/A | 3.1 (1.4) | 2.8 (1.4) | <0.001 |
| Maternal depression  (% depressed)^4^ | 20.4 | 7.8 | <0.001 | 13.1 | 11.2 | 0.01 | N/A | N/A | N/A |

Continuous variables are presented as mean (SD), categorical variables as percentages.

^1^ P-values are from t-tests for continuous variables and chi-squared tests for categorical variables in ALSPAC and Pelotas. In HUNT, P-values come from linear (continuous outcome variables) or logistic (binary outcome variables) regressions with robust standard errors clustered by the mother's identity.

^2^ In ALSPAC, this is the proportion of mothers with A-levels or higher.

^3^ Percentage of individuals in the highest category of crowding (ALSPAC >1 person/room, Pelotas> 4 people/room)

^4^ In ALSPAC, this is the proportion of mothers scoring 13 or above on the Edinburgh Post-natal Depression Scale, measured at 18 weeks gestation. In HUNT, this is the proportion of mothers categorised as depressed at survey using the Hospital Anxiety and Depression Scale.

**eTable 2. Covariates stratified by partner smoking during pregnancy in complete case analysis**

|  | **ALSPAC** | | | **HUNT** | | | **Pelotas 1982** | | |
| --- | --- | --- | --- | --- | --- | --- | --- | --- | --- |
|  | Yes | No | P-value^1^ | Yes | No | P-value^1^ | Yes | No | P-value^1^ |
| N (%) | 850 (29.6) | 2,019 (70.4) |  | 8,907 (57.5) | 6,586 (42.5) |  | 1538 (58.6) | 1088 (41.4) |  |
| Offspring age (years) | 17.8 (4.0) | 17.8 (4.0) | 0.28 | 33.2 (8.4) | 31.3 (8.8) | <0.001 | 30.2 (0.3) | 30.2 (0.3) | 0.15 |
| % male | 43.0 | 45.2 | 0.23 | 48.1 | 47.7 | 0.620 | 50.9 | 44.9 | 0.002 |
| Maternal age at birth of offspring | 28.7 (4.6) | 29.8 (4.2) | <0.001 | 25.8 (5.1) | 26.9 (5.2) | <0.001 | 26.3 (6.4) | 26.8 (6.1) | 0.07 |
| Parity | 1.7 (0.8) | 1.7 (0.8) | 0.67 | 1.7 (1.0) | 1.7 (0.9) | 0.34 | 2.7 (2.0) | 2.5 (1.9) | 0.02 |
| Social class  (% non-manual) | 55.1 | 71.7 | <0.001 | 28.3 | 32.8 | <0.001 | 14.2 | 17.6 | 0.02 |
| Maternal education  (% >12 years education)^2^ | 40.1 | 54.3 | <0.001 | 8.2 | 17.8 | <0.001 | 11.8 | 18.8 | <0.001 |
| Crowding^3^ | 4.0 | 1.4 | <0.001 | N/A | N/A | N/A | 3.0 (1.3) | 2.8 (1.4) | <0.001 |
| Maternal depression  (% depressed)^4^ | 14.1 | 7.7 | 0.001 | 13.1 | 10.2 | <0.001 | N/A | N/A | N/A |

Sample for each study is the full sample- i.e. all those who have the data required for the analysis of offspring depression and parental smoking during pregnancy, but don't necessarily have data on each potential confounder. Continuous variables are presented as mean (SD), categorical variables as percentages.

^1^ P-values are from t-tests for continuous variables and chi-squared tests for categorical variables in ALSPAC and Pelotas. In HUNT, P-values come from linear (continuous outcome variables) or logistic (binary outcome variables) regressions with robust standard errors clustered by the partner's identity.

^2^ In ALSPAC, this is the proportion of mothers with A-levels or higher.

^3^ Percentage of individuals in the highest category of crowding (ALSPAC >1 person/room, Pelotas> 4 people/room)

^4^ In ALSPAC, this is the proportion of mothers scoring 13 or above on the Edinburgh Post-natal Depression Scale, measured at 18 weeks gestation. In HUNT, this is the proportion of mothers categorised as depressed at survey using the Hospital Anxiety and Depression Scale.

**eTable 3. Covariates stratified by depression in complete case analysis**

|  | **ALSPAC** | | | **HUNT** | | | **Pelotas 1982** | | |
| --- | --- | --- | --- | --- | --- | --- | --- | --- | --- |
|  | Yes | No | P-value^1^ | Yes | No | P-value^1^ | Yes | No | P-value^1^ |
| N (%) | 199 (6.9) | 2,670 (93.1) |  | 1,205 (7.8) | 14,288 (92.2) |  | 202 (7.7%) | 2,424 (93.7%) |  |
| Offspring age (years) | 17.8 (4.0) | 17.8 (4.0) | 0.91 | 35.2 (8.7) | 32.1 (8.6) | <0.001 | 30.2 (0.3) | 30.2 (0.3) | 0.36 |
| N (%) male | 26.1 | 46.1 | <0.001 | 51.2% | 47.6% | 0.02 | 25.7 | 50.3 | <0.001 |
| Maternal age at birth of offspring | 28.8 (4.8) | 29.5 (4.3) | 0.01 | 26.0 (5.2) | 26.3 (5.2) | 0.08 | 25.4 (6.1) | 26.6 (6.2) | 0.01 |
| Parity | 1.7 (0.9) | 1.7 (0.8) | 0.12 | 1.7 (1.0) | 1.7 (0.9) | 0.54 | 2.6 (1.8) | 2.6 (2.0) | 0.92 |
| Social class  (% non-manual) | 63.8 | 67.0 | 0.36 | 28.4% | 30.4% | 0.17 | 9.9 | 16.1 | 0.02 |
| Maternal education  (% >12 years education)^2^ | 43.7 | 50.6 | 0.06 | 8.9% | 12.6% | <0.001 | 7.4 | 15.3 | 0.02 |
| Crowding^3^ | 3.5 | 2.1 | 0.19 | N/A | N/A | N/A | 3.1 (1.3) | 2.9 (1.4) | 0.11 |
| Maternal depression  (% depressed)^4^ | 14.1 | 9.3 | 0.02 | 19.0% | 11.3% | <0.001 | N/A | N/A | N/A |

Sample for each study is the full sample- i.e. all those who have the data required for the analysis of offspring depression and parental smoking during pregnancy, but don't necessarily have data on each potential confounder. Continuous variables are presented as mean (SD), categorical variables as percentages.

^1^ P-values are from t-tests for continuous variables and chi-squared tests for categorical variables in ALSPAC and Pelotas. In HUNT, P-values come from linear (continuous outcome variables) or logistic (binary outcome variables) regressions with robust standard errors clustered by the mother's identity.

^2^ In ALSPAC, this is the proportion of mothers with A-levels or higher.

^3^ Percentage of individuals in the highest category of crowding (ALSPAC >1 person/room, Pelotas> 4 people/room)

^4^ In ALSPAC, this is the proportion of mothers scoring 13 or above on the Edinburgh Post-natal Depression Scale, measured at 18 weeks gestation. In HUNT, this is the proportion of mothers categorised as depressed at survey using the Hospital Anxiety and Depression Scale.

**eTable 4.** Discordant sibling analysis in Swedish Sibling Health Cohort

|  | **N individuals** | **N families** | **Mean age of offspring (SD)** | **Male (%)** | **Unadjusted** | | **Fully adjusted^1^** | |
| --- | --- | --- | --- | --- | --- | --- | --- | --- |
|  |  |  |  |  | **OR** | **(95% CI)** | **OR** | **(95% CI)** |
| **Swedish sibling health study** | 516 | 258 | 24.2 (2.5) | 28.7 | 1.12 | (0.87, 1.42) | 1.03 | (0.77,1.36) |

^1^ Swedish Sibling Health Cohort analyses adjusted for maternal age, calendar period at birth, parity, sibling order.

**eTable 5. Prevalence of maternal and partner smoking and offspring depression in individuals included and excluded from final analysis sample**

|  | **Maternal smoking during pregnancy (%)** | | | **Partner smoking during pregnancy (%)** | | | **Offspring depression (%)** | | |
| --- | --- | --- | --- | --- | --- | --- | --- | --- | --- |
|  | **In final analysis sample** | **Not in final analysis sample** | **P-value** | **In final analysis sample** | **Not in final analysis sample** | **P-value** | **In final analysis sample** | **Not in final analysis sample** | **P-value** |
| **ALSPAC^1^** | 14.3% | 34.1% | <0.001 | 29.6% | 43.7% | <0.001 | 6.9% | 9.7 % | 0.001 |
| **HUNT^2^** | 36.0% | 25.9% | <0.001 | 57.5% | 57.0% | 0.507 | 8.0% | 13.4% | <0.001 |
| **Pelotas 1982^3^** | 33.2% | 37.5% | 0.001 | 58.6% | 59.0% | 0.81 | 7.7% | 8.6% | 0.36 |

^1^ Based on N of 12,999 for maternal smoking, 13,125 for partner smoking and 4,441 for depression

^2^ Based on N of 40,582 for maternal smoking, 34,161 for partner smoking and 38,163 for depression

^3^ Based on N of 5,914 for maternal smoking, 4.105 for partner smoking and 3,576 for depression

**eTable 6. Associations between parental smoking during pregnancy and offspring depression after multiple imputation of missing data**

|  | **N** | **Unadjusted**  **OR (95% CI)** | **P-value** | **Partially adjusted**  **OR (95% CI) ^1^** | **P-value** | **Fully adjusted**  **OR (95% CI) ^2^** | **P-value** | **Mutually adjusted**  **OR (95% CI) ^3^** | **P-value** |
| --- | --- | --- | --- | --- | --- | --- | --- | --- | --- |
| **ALSPAC (imputed to individuals with exposure data)^4^** | | | | | | | | | |
| Maternal smoking | 8,105 | 1.61 (1.26, 2.06) | <0.001 | 1.63 (1.28, 2.09) | <0.001 | 1.41 (1.07, 1.86) | 0.02 | 1.50 (1.12, 2.01) | 0.007 |
| Partner smoking | 8,105 | 1.11 (0.87, 1.40) | 0.40 | 1.09 (0.86, 1.39) | 0.46 | 0.94 (0.73, 1.20) | 0.58 | 0.84 (0.65, 1.09) | 0.19 |
| **ALSPAC (imputed to individuals with CIS-R outcome data)^5^** | | | | | | | | | |
| Maternal smoking | 4,441 | 1.74 (1.34, 2.26) | <0.001 | 1.76 (1.35, 2.29) | <0.001 | 1.50 (1.12, 2.01) | 0.006 | 1.60 (1.17, 2.17) | 0.003 |
| Partner smoking | 4,441 | 1.13 (0.89, 1.43) | 0.30 | 1.11 (0.88, 1.41) | 0.39 | 0.95 (0.74, 1.22) | 0.68 | 0.84 (0.64, 1.10) | 0.21 |
| **HUNT (imputed to all individuals with at least one participating parent)^5^** | | | | | | | | | |
| Maternal smoking | 45,988 | 0.71 (0.65, 0.77) | <0.001 | 1.10 (1.00, 1.20) | 0.032 | 1.05 (0.96, 1.15) | 0.318 | 1.06 (0.97, 1.16) | 0.202 |
| Partner smoking | 45,988 | 0.97 (0.90, 1.05) | 0.454 | 0.99 (0.92, 1.07) | 0.845 | 0.94 (0.87, 1.02) | 0.157 | 0.94 (0.86, 1.02) | 0.117 |

**^1^** Adjusted for offspring age and sex.

**^2^** Adjusted for all covariates. ALSPAC: maternal age, partner social class, maternal education, maternal and paternal antenatal depression and anxiety, parity, housing tenure, crowding. HUNT: maternal age, partner occupation, maternal education, maternal and paternal depression and anxiety at survey, parity, wave of HUNT participation, number of HUNT participations. Pelotas 1982: maternal age, social class, maternal education, household income, assets index, crowding.

**^3^** Adjusted for all covariates and the other parent’s smoking.

^4^ Prevalence of depression: 8.3%

^5^ Prevalence of maternal smoking: 18%, Prevalence of paternal smoking: 33%

^6^ Prevalence of depression: 11.3%, prevalence of maternal smoking: 28.4%, prevalence of paternal smoking: 57.0%

ALSPAC results imputed 100 times using the mi impute command in Stata: maternal smoking during pregnancy, paternal smoking during pregnancy, CIS-R depression, Moods and feelings questionnaire data (6 different timepoints), paternal social class, maternal social class, maternal education, maternal depression during pregnancy (EPDS), maternal depression just post birth (EPDS), maternal anxiety during pregnancy, parity, crowding, tenure, maternal age, maternal alcohol consumption during pregnancy, partner alcohol consumption during pregnancy, partner depression during pregnancy, impulsivity, homeowner, neighbourhood, dwelling type, breastfeeding at 12 months, household income, paternal anxiety during pregnancy, sex, offspring age, offspring smoking, offspring BMI, paternal education, maternal BMI pre pregnancy, birth weight, gestational age, dysfunctional questionnaire at age 18 years.

HUNT results imputed 100 times using the mi impute command in Stata: maternal BMI, paternal BMI, offspring BMI, maternal age at offspring birth, offspring age at participation, maternal smoking during pregnancy, paternal smoking during pregnancy, maternal depression, paternal depression, offspring depression, maternal anxiety, paternal anxiety, offspring anxiety, offspring smoking at 17, maternal non-manual employment (binary), offspring non-manual employment (binary), offspring sex, offspring participation round, parity (four categories), maternal education (3 categories), paternal education, offspring education, maternal alcohol consumption (3 categories), paternal alcohol consumption, offspring alcohol consumption, maternal physical activity (3 categories), paternal physical activity, offspring physical activity, paternal employment (7 categories).

**eTable 7. Associations between parental smoking and offspring depression, with individuals with data on maternal depression in Pelotas 1982**

|  | **N** | **Unadjusted**  **OR (95% CI)** | **P-value** | **N** | **Partially adjusted**  **OR (95% CI) ^1^** | **P-value** | **N** | **Fully adjusted**  **OR (95% CI) ^2^** | **P-value** | **N** | **Mutually adjusted**  **OR (95% CI) ^3^** | **P-value** |
| --- | --- | --- | --- | --- | --- | --- | --- | --- | --- | --- | --- | --- |
| Maternal  smoking | 886 | 1.27  (0.78 - 2.06) | 0.29 | 886 | 1.22  (0.75 - 1.99) | 0.37 | 886 | 1.05  (0.64 - 1.73) | 0.83 | 886 | 1.05  (0.64 - 1.75) | 0.80 |
| Partner  smoking | 886 | 1.20  (0.74 - 1.95) | 0.49 | 886 | 1.21  (0.74 - 1.97) | 0.47 | 886 | 1.15  (0.70 - 1.89) | 0.76 | 886 | 1.14  (0.69 - 1.90) | 0.79 |

Maternal depression was assessed when the offspring were 18/19 years by the self-rating questionnaire (SRQ) for depression ^14^

**^1^** Adjusted for offspring age and sex.

**^2^** Adjusted for Pelotas 1982: maternal age, social class, maternal education, household income, assets index, crowding and maternal depression.

**^3^** Adjusted for all covariates and the other parent’s smoking.

**References**

1. Boyd A, Golding J, Macleod J, et al. Cohort Profile: The 'Children of the 90s'--the index offspring of the Avon Longitudinal Study of Parents and Children. *International journal of epidemiology.* 2013;42(1):111-127.

2. Fraser A, Macdonald-Wallis C, Tilling K, et al. Cohort Profile: The Avon Longitudinal Study of Parents and Children: ALSPAC mothers cohort. *International journal of epidemiology.* 2013;42(1):97-110.

3. Lewis G, Pelosi AJ, Araya R, Dunn G. Measuring psychiatric disorder in the community: a standardized assessment for use by lay interviewers. *Psychological medicine.* 1992;22(2):465-486.

4. Cox JL, Holden JM, Sagovsky R. Detection of postnatal depression. Development of the 10-item Edinburgh Postnatal Depression Scale. *The British journal of psychiatry : the journal of mental science.* 1987;150:782-786.

5. Crown S, Crisp AH. A short clinical diagnostic self-rating scale for psychoneurotic patients. The Middlesex Hospital Questionnaire (M.H.Q.). *The British journal of psychiatry : the journal of mental science.* 1966;112(490):917-923.

6. Bjelland I, Dahl AA, Haug TT, Neckelmann D. The validity of the Hospital Anxiety and Depression Scale: An updated literature review. *Journal of Psychosomatic Research.* 2002;52(2):69-77.

7. Harrell FE. *Regression Modeling Strategies. With applications to linear models, logistic regression, and survival analysis.* New York: Springer; 2001.

8. Horta BL, Gigante DP, Goncalves H, et al. Cohort Profile Update: The 1982 Pelotas (Brazil) Birth Cohort Study. *International journal of epidemiology.* 2015;44(2):441, 441a-441e.

9. Barros AJ, Santos IS, Matijasevich A, et al. Methods used in the 1982, 1993, and 2004 birth cohort studies from Pelotas, Rio Grande do Sul State, Brazil, and a description of the socioeconomic conditions of participants' families. *Cad Saude Publica.* 2008;24 Suppl 3:S371-380.

10. Sichieri R, Benicio MH, Barreto SM, Lima-Costa MF. [The 1982 Pelotas birth cohort]. *Rev Saude Publica.* 2008;42 Suppl 2:1-2.

11. Amorim P. Mini International Neuropsychiatric Interview (MINI): validação de entrevista breve para diagnóstico de transtornos mentais. *Revista Brasileira de Psiquiatria.* 2000;22:106-115.

12. Rydell M, Granath F, Cnattingius S, Magnusson C, Galanti MR. In-utero exposure to maternal smoking is not linked to tobacco use in adulthood after controlling for genetic and family influences: a Swedish sibling study. *European journal of epidemiology.* 2014;29(7):499-506.

13. *The Swedish medical birth register: a summary of content and quality. .* Stockholm, Sweden2003.

14. Goncalves H, Pearson RM, Horta BL, et al. Maternal depression and anxiety predicts the pattern of offspring symptoms during their transition to adulthood. *Psychological medicine.* 2015:1-10.
